# Mixed IgG Fc immune complexes exhibit blended binding profiles and refine FcR affinity estimates

**DOI:** 10.1016/j.celrep.2023.112734

**Published:** 2023-07-07

**Authors:** Zhixin Cyrillus Tan, Anja Lux, Markus Biburger, Prabha Varghese, Stephen Lees, Falk Nimmerjahn, Aaron S. Meyer

**Affiliations:** 1 Bioinformatics Interdepartmental Program, University of California, Los Angeles (UCLA), Los Angeles, CA 90095, USA; 2 Division of Genetics, Department of Biology, Friedrich-Alexander-Universität Erlangen-Nürnberg, 91058 Erlangen, Germany; 3 Department of Bioengineering, UCLA, Los Angeles, CA 90095, USA; 4 Jonsson Comprehensive Cancer Center, UCLA, Los Angeles, CA 90095, USA; 5 Eli and Edythe Broad Center of Regenerative Medicine and Stem Cell Research, UCLA, Los Angeles, CA 90095, USA; 6 These authors contributed equally; 7 Lead contact

## Abstract

Immunoglobulin G (IgG) antibodies coordinate immune effector responses by interacting with effector cells via fragment crystallizable γ (Fcγ) receptors. The IgG Fc domain directs effector responses through subclass and glycosylation variation. Although each Fc variant has been extensively characterized in isolation, during immune responses, IgG is almost always produced in Fc mixtures. How this influences effector responses has not been examined. Here, we measure Fcγ receptor binding to mixed Fc immune complexes. Binding of these mixtures falls along a continuum between pure cases and quantitatively matches a mechanistic model, except for several low-affinity interactions mostly involving IgG2. We find that the binding model provides refined estimates of their affinities. Finally, we demonstrate that the model predicts effector cell-elicited platelet depletion in humanized mice. Contrary to previous views, IgG2 exhibits appreciable binding through avidity, though it is insufficient to induce effector responses. Overall, this work demonstrates a quantitative framework for modeling mixed IgG Fc-effector cell regulation.

## In brief

Tan et al. quantify the binding of IgG immune complexes with mixed Fc domains to the Fcγ receptors, refine estimates of FcγR-IgG affinities with a computational binding model, and then demonstrate that this model can predict effector cell-elicited platelet depletion in humanized mice.

## Graphical Abstract

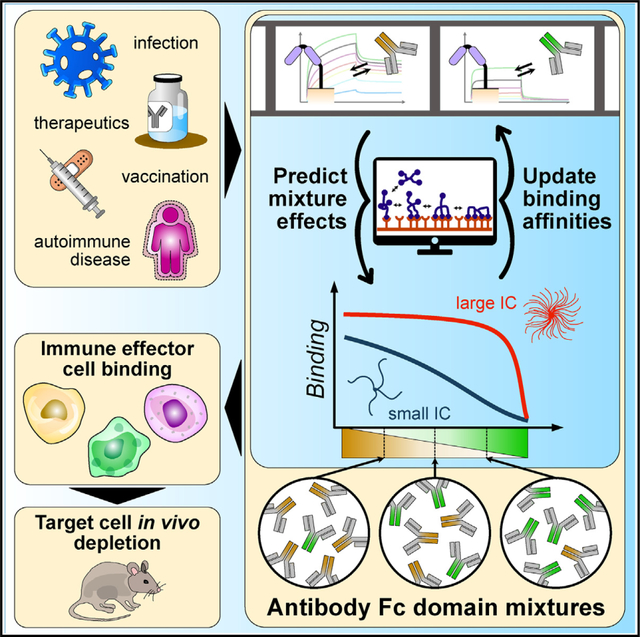

## INTRODUCTION

Antibodies are both a core component of adaptive immunity and a versatile platform for developing therapies. An antibody’s role in promoting immunity is defined by its selectivity toward a target antigen, as determined by its variable region, and its ability to elicit effector cell responses, defined by the composition of its constant, fragment crystallizable (Fc) region. Antibodies of the immunoglobulin G (IgG) type direct effector responses by binding to Fcγ receptors (FcγRs) via their Fc region. FcγR activation is initiated through IgG-mediated clustering, which in turn is caused by the engagement of several antibodies on an antigen target, forming an immune complex (IC). Depending upon the receptors included, this interaction may promote or prevent an effector response. This clustering mechanism ensures that more than one IgG is present whenever effector responses occur.

The immune response triggered by an IgG IC consisting of a specific Fc form, including subclass or glycosylation, is defined by its binding to specific FcγRs, each of which differs in signaling effect and expression patterns.^1^ Consequently, accurate estimates of IgG Fc-FcγR affinities are essential to understanding their effect. Most existing FcγR affinity measurements have been performed by surface plasmon resonance (SPR) using monovalent IgG.^2,3^ SPR accurately assesses protein-protein binding kinetics, but many antibody-Fc receptor interactions are weak enough to fall outside the assay’s quantitative range when assessed in monovalent form. Clustering leads to avidity effects wherein even weak interactions can cooperatively lead to strong binding.^4^ Indeed, avidity is widely employed in natural and engineered systems to promote binding through low-affinity interactions.^5^ Therefore, direct measurement of IC binding might more accurately quantify IgG Fc properties, particularly for low-affinity interactions. Measuring Fc binding as multivalent ICs additionally resembles the relevant *in vivo* context of effector responses.^6^

Physiological antibody responses universally involve Fc mixtures. For instance, during the course of infection, the composition of IgG subclasses shifts dynamically to different subclasses due to class switching.^7^ Even when recombinantly manufacturing monoclonal therapeutic antibody preparations, heterogeneity exists in the glycosylation forms derived, and this glycan heterogeneity likely exists during endogenous antibody production as well.^8,9^ With mixtures of antibodies of varied Fc composition but identical antigen binding, there might be an additive combination of effects, or a minor species (e.g., glycosylation variant) might present an outsized effect promoting or preventing effector responses. Therefore, knowledge of how these different forms influence the behavior of one another would allow one to modulate immune responses by adjusting subclass composition. With respect to therapeutic monoclonal antibody preparations, this would help guide the evaluation of biosimilars by determining whether glycosylation forms present at small fractions influence overall therapeutic efficacy.^10^

After binding to Fc receptors, effector cell-elicited responses to IgG include several different functionally distinct mechanisms, including antibody-dependent cell cytotoxicity (ADCC) and phagocytosis (ADCP). Effector responses are coordinately regulated by the cell types present within a tissue,^11,12^ the FcγRs expressed on those effector cells,^13^ the Fc regions present within an IC,^1^ and the properties of antigen engagement.^14,15^ Regulation at the Fc receptor and cell population levels is a challenge to engineering antibodies with desirable cell-killing functions, as well as understanding both productive and pathogenic immune responses. Furthermore, it has become clear that, in addition to natural killer (NK) cells, tissue-resident macrophages and bone marrow-derived monocytes participate in cytotoxic antibody-dependent target cell clearance. In contrast to NK cells (expressing only one activating FcγR, FcγRIIIA), these myeloid cell subsets express a broader set of activating FcγRs and the inhibitory FcγRIIB.^13^ Thus, a mixed IC may trigger all or specific subsets of activating/inhibitory FcγRs, resulting in further complexity. Despite the presence of and capacity to bind to multiple activating FcγRs on myeloid effector cells, our previous studies have demonstrated that individual IgG subclasses, such as mIgG2a/c, may mediate their activity through select activating FcγRs, indicating that there may be specialization in FcγR signaling.^6^

Our team recently demonstrated that a model of IC-FcγR binding accurately captured and could predict *in vitro* binding across various IgG isotypes.^16^ Further, it could accurately predict antibody-elicited tumor cell killing in mice across antibodies of varied isotypes, glycosylation statuses, and FcγR knockouts.^16^ Directly quantifying and predicting cell clearance makes it possible to accurately anticipate and optimize for antibody-mediated therapeutic effects. However, it is still unclear whether such a modeling strategy can accurately predict the response of human immune cells, particularly given the divergent properties between the murine and human receptors,^17–19^ and whether this modeling strategy can extend to ICs of mixed composition.

Here, we examined the binding properties of ICs with mixed IgG Fc composition. We quantified the binding of these ICs to each individual FcγR and observed that mixed-composition ICs resulted in a continuum of binding responses. A multivalent binding model extended to heterovalent IC mixtures captured binding overall. However, surprisingly, it did not match certain low-affinity interactions.^20^ Investigating the source of this discrepancy allowed us to improve the estimates of these interactions’ affinities. We additionally demonstrate that the binding model can be used to both predict *in vivo* effector responses in humanized mice and infer the cell types responsible for these responses. Thus, while antibody effector responses operate through a complex milieu of antibody species, Fc receptors, and cell types, IC profiling paired with modeling provides a framework to reason about the role of each molecular and cellular element.

## RESULTS

### Profiling the binding effects of mixed-composition ICs

**Figure 1.**
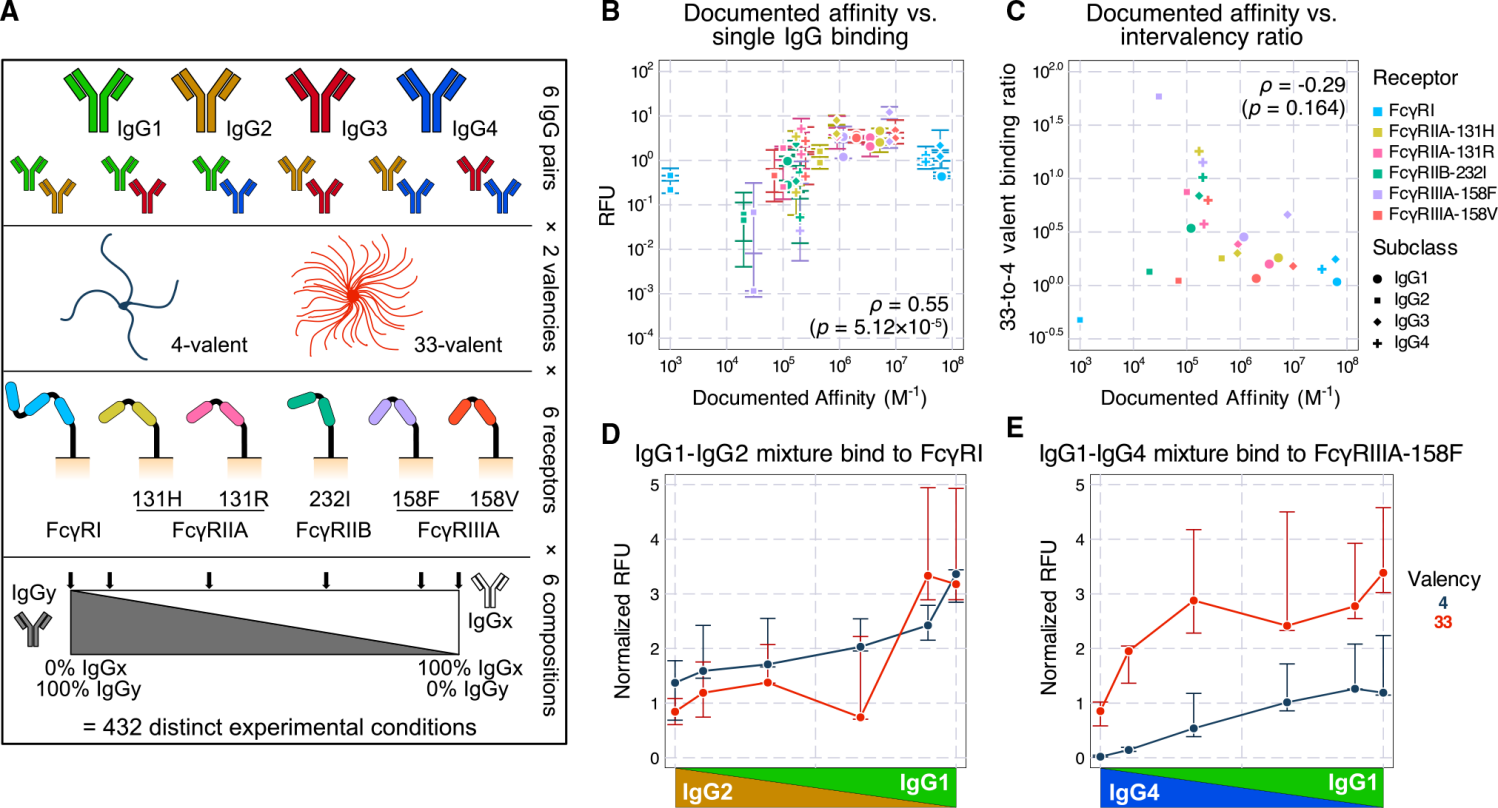
Profiling the binding effects of mixed-composition immune complexes (A) Schematic of the immune complex (IC) binding experiment. Individual or mixtures of IgG subclasses are immobilized as multivalent TNP complexes. The binding of these complexes to CHO cells expressing a single type of Fc receptor is then quantified. (B) Measured binding in relative fluorescence units (RFUs) versus the previously reported affinity of each interaction. Only single subclass conditions are plotted. Each condition has 3–5 technical replicates. Error bars represent the interquartile range of the measurements. (C) The ratio of median binding quantified between valencies 33 and 4 versus the reported affinity of the interaction. ρ represents the Spearman correlation coefficient. Significance testing was performed using the t-statistic under the null hypothesis that ρ = 0. (D) IgG1-IgG2 mixture binding to FcγRI shows appreciable binding even though IgG2-FcγRI is documented to be non-binding. The RFU level was normalized to match the FcγRI expression to the FcγRIIIA-158F expression (shown in E) on CHO cells. (E) IgG1-IgG4 mixture binding to FcγRIIIA-158F. In (D) and (E), each error bar represents the interquartile range of the three technical replicates in the respective condition.

To determine the effect of having multiple Fc forms present within an IC, we developed a controlled and simplified *in vitro* system. Like in previous work, we employed a panel of CHO cell lines expressing one of six individual human FcγRs^16^ (Figure 1A). ICs were formed by immobilizing anti-2,4,6-trinitrophenol (TNP) human IgG on conjugates of TNP and bovine serum albumin (TNP-BSA) with an average valency of 4 or 33. IgG binding was then quantified after incubation with the cells using a constant IC concentration of 1 nM (Figure S1). In contrast to our previous work using a single IgG isotype, we assembled ICs from mixtures of each IgG isotype pair.^16^ For each pair of IgGs, ICs were formed with a spectrum of six compositions of the IgG pair, including 100%/0%, 90%/10%, and 67%/33% mixtures. Combinations of 6 FcγRs, 2 valencies, 6 IgG pairs, and 6 IgG compositions resulted in 432 distinct experimental conditions. One-way ANOVA showed that more than 70% variance in the data was between experimental conditions rather than within them, indicating that more than 70% of the variance could be explained by biological differences (Table S1). This suggests that, within each condition, measurements were consistent.

Inspection of the resulting binding data revealed several expected patterns. Among the conditions with only one IgG present, the measured binding showed a strong, positive correlation with the documented IgG-FcγR interaction affinities (Figure 1B). The higher-valency ICs universally showed greater binding signals compared with their matching lower-valency counterparts, and there is an obvious negative trend between documented affinities and the ratio between the 33-valent and 4-valent complex binding (Figure 1C). This trend is expected since, although complexes of both valencies can bind densely with high-affinity units, only high-valent complexes compensate for low affinity through avidity.^4^ Therefore, while high-affinity complexes result in greater binding, low-affinity complexes have greater intervalency binding ratios. Finally, mixtures spanning 100% of one IgG isotype to another generally showed a monotonic shift with composition (Figure S1). These patterns, along with their reproducibility (Table S1), gave us confidence in the quality of the binding measurements.

We also observed several unexpected trends among the binding measurements. There was appreciable binding from IgG2-FcγRI interactions despite this combination being reported as non-binding^3^ (Figure 1D). We also saw an increase in binding along the shift from IgG4 to IgG1 with FcγRIIIA-158F, even though these two isotypes are documented to have identical affinities^3^ (Figure 1E). These two observations are consistent with previous binding measurements using the same TNP-based IC system.^16^

**Figure 2.**
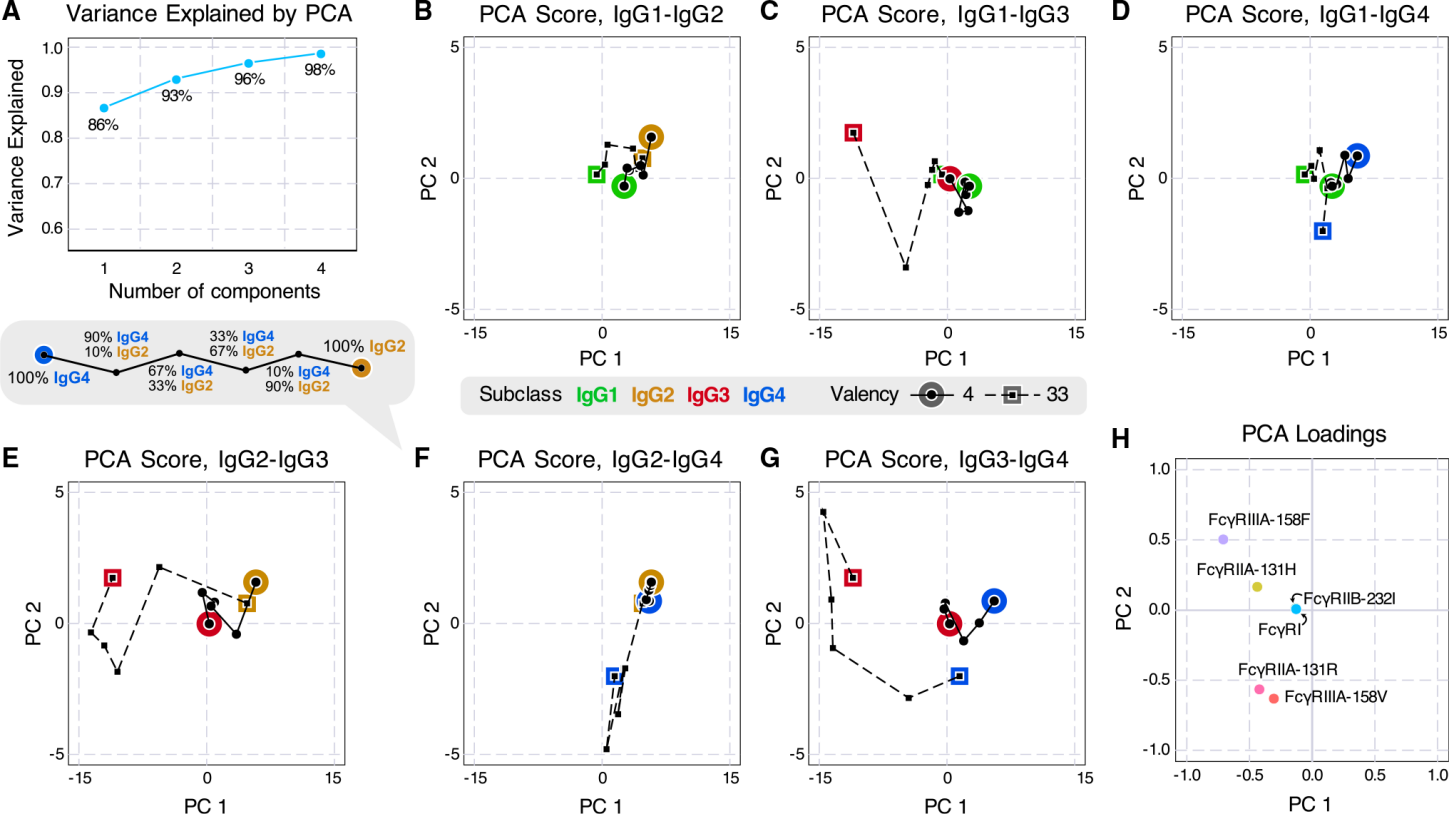
Principal component analysis (PCA) visualizes the variance in mixture binding measurements and their associated factors (A) Variance explained by each number of components in PCA. Two PCs explained greater than 93% of the measurement variance. (B–G) PCA scores for ICs of each valency and pair of IgG subclasses. (H) PCA loadings. The FcγRI and FcγRIIB-232I points overlap.

To better visualize the binding of these experimental conditions, we performed principal component analysis (PCA) on the median measurement of each condition, with each isotype mixture and valency as a sample and each receptor as a feature. The first principal component (PC1) explains more than 86% of the variance, and the first two components (PC1 and PC2) explain 93% (Figure 2A). Inspecting the scores, we found that the 33-valent measurements are more broadly distributed, consistent with their greater expected binding (Figures 2B–2G). PC1 mostly separates IgG3 binding from other isotypes, reflecting that IgG3 has the greatest binding among IgG subclasses (Figure S1). PC2 separated the genotype variants of FcγRIIA and FcγRIIIA and associated most strongly with IgG3 and IgG4 (Figure 2H), reflecting that these two subclasses showed larger differences in binding with genotype (Figure S1).

In all, these data support that TNP-assembled ICs provide a controlled *in vitro* system in which we can profile the effects of mixed IC composition on binding to effector cell populations. Quantifying binding using ICs may, in fact, provide more precise quantification of IgG-FcγR interaction affinities, particularly for lower-affinity pairs, and mixed Fc composition ICs showed binding between that of the corresponding single Fc cases.

### A multivalent binding model accurately predicts *in vitro* IgG mixture binding and updates Fc-FcγR affinities

**Figure 3.**
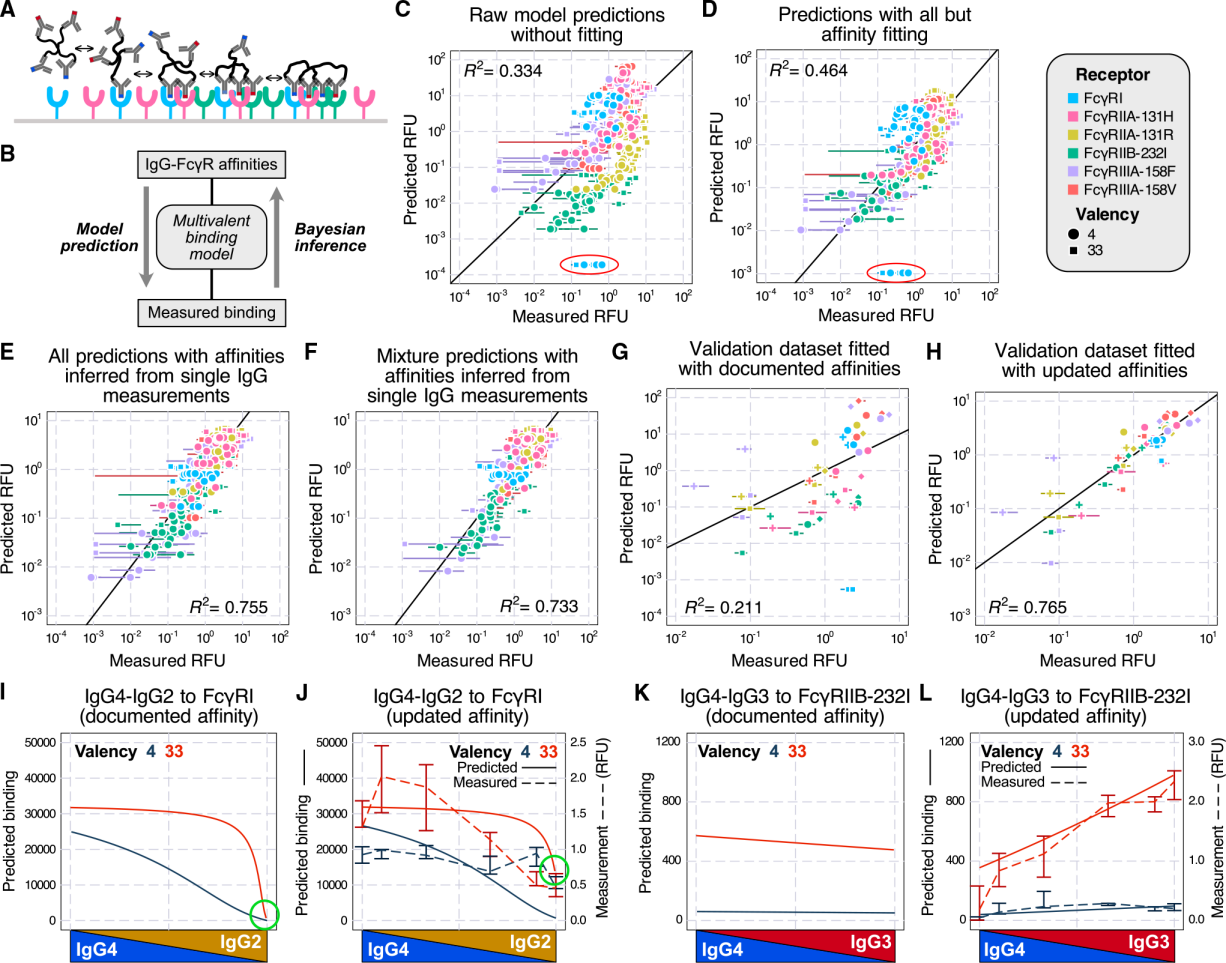
A multivalent binding model accurately accounts for *in vitro* binding of IgG mixtures (A) Schematic of the multivalent binding model. (B) Schematic of the process of predicting binding with documented affinities and inferring affinities from measurements. (C) Measured versus predicted binding by the binding model without fitting. Points also vary in the IgG subclass used, which is not indicated. (D) Binding model prediction with all parameters but affinities fitted by Markov chain Monte Carlo (MCMC). In (C) and (D), the IgG2-FcγRI outliers are circled in red. Since this interaction was previously reported as non-binding, the actual predictions were all 0 but were clipped to a non-zero value (1/10 of the next smallest value) to be plotted on the log scale. (E) Binding model prediction of all measurements (single and mixed IgG) with affinity inferred from the single IgG measurements. (F) Binding model prediction of mixture IgG measurements with affinities updated using the single IgG measurements. In (C)–(F), error bars represent the interquartile range of 3–5 technical replicates. (G and H) Validation of the updated affinities with a separate dataset^16^ by predicting the binding with either documented (G) or updated (H) affinities. The error bars represent the interquartile range of four technical replicates for each condition. (I–L) Predicted binding of IgG4-IgG2 mixture to FcγRI (I and J) and IgG4-IgG3 mixture to FcγRIIB-232I (K and L) with either documented (I and K) or updated affinities (J and L, solid line and left axis) compared with measured binding (J and L, dashed line and right axis). Error bars in (J) and (L) represent the interquartile range of 3–5 technical replicates.

To model the effects of polyclonal antibody responses, we extended a simple, equilibrium-binding model that we have previously used to model antibody effector response.^16,20^ Briefly, ICs are assumed to bind to FcγRs on the cell surface with monovalent binding kinetics and then can engage additional receptors with a propensity proportional to their affinity (Figure 3A). Though additional assumptions are not required for modeling ICs of mixed isotype composition, this extension leads to a large combinatorial expansion in the number of binding configurations. Through some properties of combinatorics, we derived simplified expressions for many macroscopic quantities to allow this model to scale to multiligand, multireceptor, and multivalent situations.^20^

We first used the measured receptor expression (Table S2) and documented affinities^3^ with the model and obtained reasonable agreement overall (Figure 3C). While the predicted values mostly agreed with the measurements, there were several notable outliers, most prominently an underestimate of IgG2-FcγRI binding (Figure 3C, red circle). To improve the measurement fit, we reversed the estimation process and used the measured binding to infer the interaction affinities via Markov chain Monte Carlo (MCMC) (Figure 3B). We first created a baseline fit quality by fitting all but the affinities (e.g., receptor abundance and the crosslinking parameter $K_{x}^{*}$; Figure 3D). Although the fit improved, outliers persisted (circled in red in Figure 3D). Therefore, we next performed the fitting while allowing the Fc-FcR affinities to vary. Although we only used the single IgG measurements to infer the Fc affinities (Figure 3E), we obtained much more accurate predictions for all measurements of both single and mixed IgG compositions (Figure 3F).

To further confirm the generality of these updated affinities, we validated these updated affinity estimates with an independent dataset collected in a previous study.^16^ This previous study independently measured the binding of TNP-BSA complexes *in vitro* with two distinct average valencies (4 and 26) but only the binding of single IgG isotypes. We set the Fc affinities to either documented or updated values and let MCMC fit the other parameters. The updated affinities resulted in a vastly improved agreement with the data (Figures 3G and 3H).

To illustrate the impact of the affinity changes, we compared the binding predictions with two sets of affinities (Figures S2 and S3) with their corresponding measurements (Figure S1). For FcγRI binding to IgG2-IgG4 mixtures, the experiment indicated that there was still notable binding with mostly or 100% IgG2, while IgG2-FcγRI was documented as non-binding.^3^ The updated values amended the prediction and reflected this interaction, especially for the 33-valent complex (Figures 3I and 3J, green circle). For FcγRIIB-232I binding to IgG3-IgG4, the documented affinities indicated that there should be more binding to IgG4 compared with IgG3, contrary to our observation (Figure 3K). The updated affinities instead accurately predicted the binding of all mixtures at both valencies (Figure 3L). These examples demonstrate that the affinity adjustments greatly improved agreement with the binding measurements.

**Figure 4.**
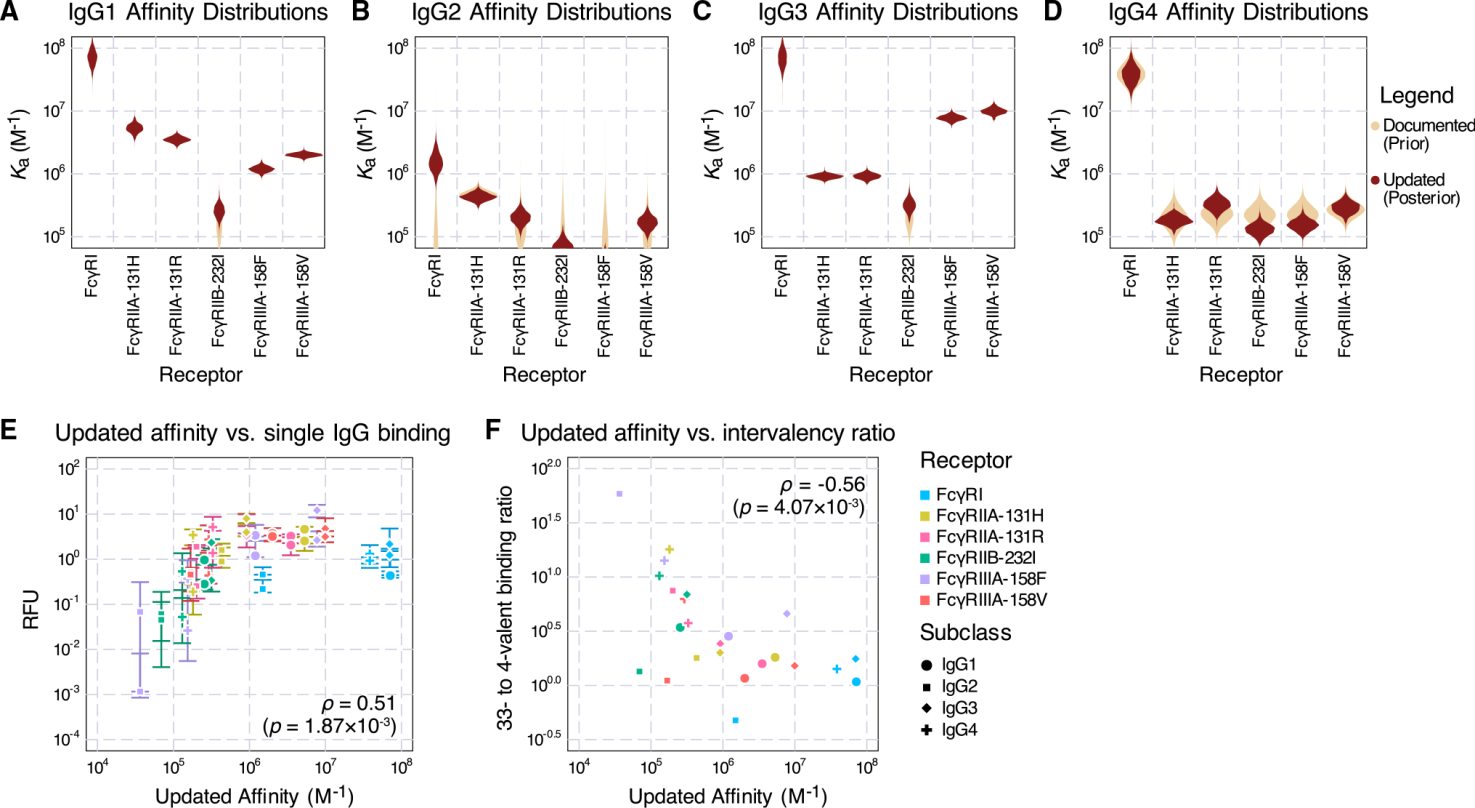
Inferred affinities from the binding data (A–D) The prior (documented) distributions of binding affinities (assuming all follow log-normal distributions) and posterior (updated) affinities of IgG1 (A), IgG2 (B), IgG3 (C), and IgG4 (D). (E) Updated affinities plot against the binding measurements of single IgGs. Error bars represent the interquartile range of the 3–5 technical replicates. (F) Updated affinities plot against the ratio of median binding between valency 33 and 4 complexes. In (E) and (F), ρ represents the Spearman correlation coefficient. Significance testing was performed using the t-statistic under the hypothesis that ρ = 0.

As our Fc affinity inference was constructed in a Bayesian fashion, both the prior (documented) and the posterior (updated) affinity values are represented as distributions accounting for uncertainty. Inspecting these updated distributions (Figures 4A–4D; Table S3), we noted several trends. The model made the largest adjustments to the Fc affinities of IgG2 (Figure 4B), followed by IgG4 (Figure 4D). Most IgG1 (Figure 4A) and IgG3 (Figure 4C) affinities remained unmodified except for a slight increase in their FcγRIIB-232I affinities. The most notable update occurred to IgG2-FcγRI. Previously reported as non-binding, FcγRI was revised to be the highest-affinity receptor for IgG2, consistent with the receptor’s high affinity to other human IgG subclasses. This discrepancy was reflected in the model prediction before affinity fitting, where the IgG2-FcγRI binding was the striking outlier (Figure 3D and 3G). Another significant adjustment occurred with IgG3-FcγRIIB-232I. Although FcγRIIB-232I has a low affinity for all IgG subclasses, our update led to IgG3 being the strongest-binding subclass (Figures 4C and S3; Table S3). More subtle differences can be observed from specific model predictions (Figure S2 and S3). The revised affinities showed a similar overall correlation with binding overall (Figure 4E). The intervalency binding ratios show a more prominent negative correlation, however, due to the movement of the IgG2-FcγRI outlier (Figure 4F).

### Multivalent binding predicts antibody-elicited effector responses in humanized mice

**Figure 5.**
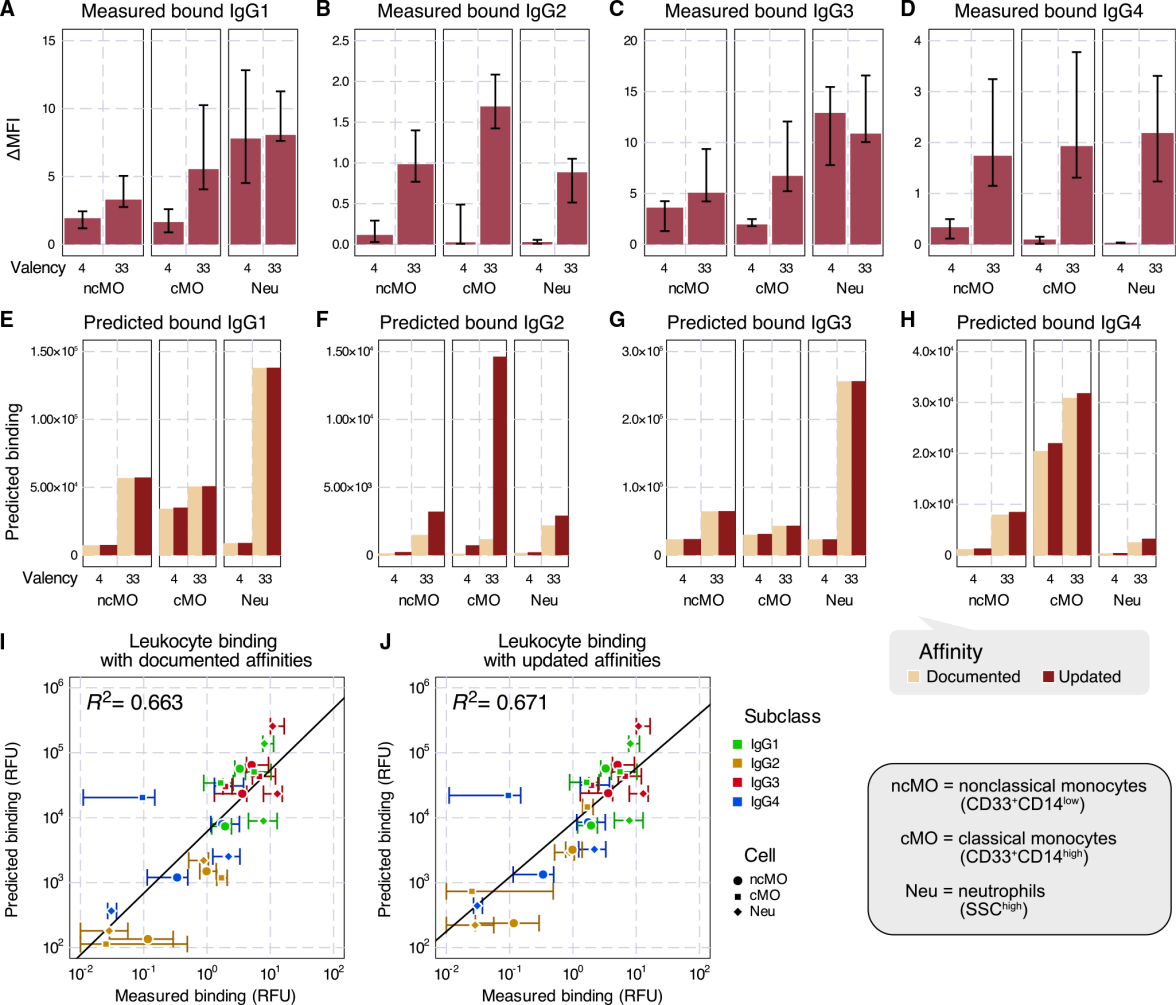
Predicting IgG effector cell binding with the multivalent binding model (A–D) Measured *in vitro* binding of IgG1 (A), IgG2 (B), IgG3 (C), and IgG4 (D) IC of either 4 or 33 valency to selective immune effector cells from human donors, classical (cMO) or non-classical monocytes (ncMO), and neutrophils (Neu). (E–H) Model-predicted IgG1 (E), IgG2 (F), IgG3 (G), and IgG4 (H) IC of 4- or 33-valent binding on each effector cell type under documented versus updated affinities. (I and J) Measured versus predicted effector leukocyte binding under documented (I) or updated (J) affinities. The error bars in (A)–(D) and (I) and (J) represent the interquartile range of six biological replicates.

We next sought to link the binding of ICs to their effects on the clearance of antigen targets *in vivo*. To quantify the antibody-driven activity of each effector cell, we first measured the binding of each human IgG subclass to immune effector cells collected from the peripheral blood of human donors *in vitro* in the ICs of two valencies, 4 and 33 (Figures 5A–5D). The measurements show that the binding amounts of IgG1 and IgG3 were generally about 10-fold higher in magnitude than those of IgG2 and IgG4. For the latter two subclasses, their 4-valent complex binding was almost negligible. In all cases except IgG2, neutrophils had more binding than classical and non-classical monocytes.

We predicted the same quantities of IC binding by the multivalent binding model with either the previously documented^3^ or updated affinities (Table S4) and the quantification of FcγR abundance^13^ (Table S6; Figure 5E–5H). These estimated binding amounts broadly aligned with the measurements (Figure 5I and 5J). Between the two sets of affinities, the predictions for IgG1 and IgG3 remained almost identical (Figure 5E and 5G), while more differences were reflected in IgG2 and IgG4 (Figure 5F and 5H), consistent with the affinities changing more for IgG2 and IgG4 (Figures 4A–4D). The predictions with documented and updated affinities were generally comparable in their concordance with the measurements (Figures 5I and 5J). However, the predicted binding to non-classical and classical monocytes was adjusted to be much higher for 33-valent IgG2 (Figure 5F), better matching the measured values (Figures 5I and 5J). Both sets of affinities predicted the binding of IgG4 to classical monocytes to be much higher than the measurements (Figures 5I and 5J). These changes indicate that the updated affinities better predict IgG IC binding to effector cells, suggesting that they may also help improve the estimation of *in vivo* cell response.

**Figure 6.**
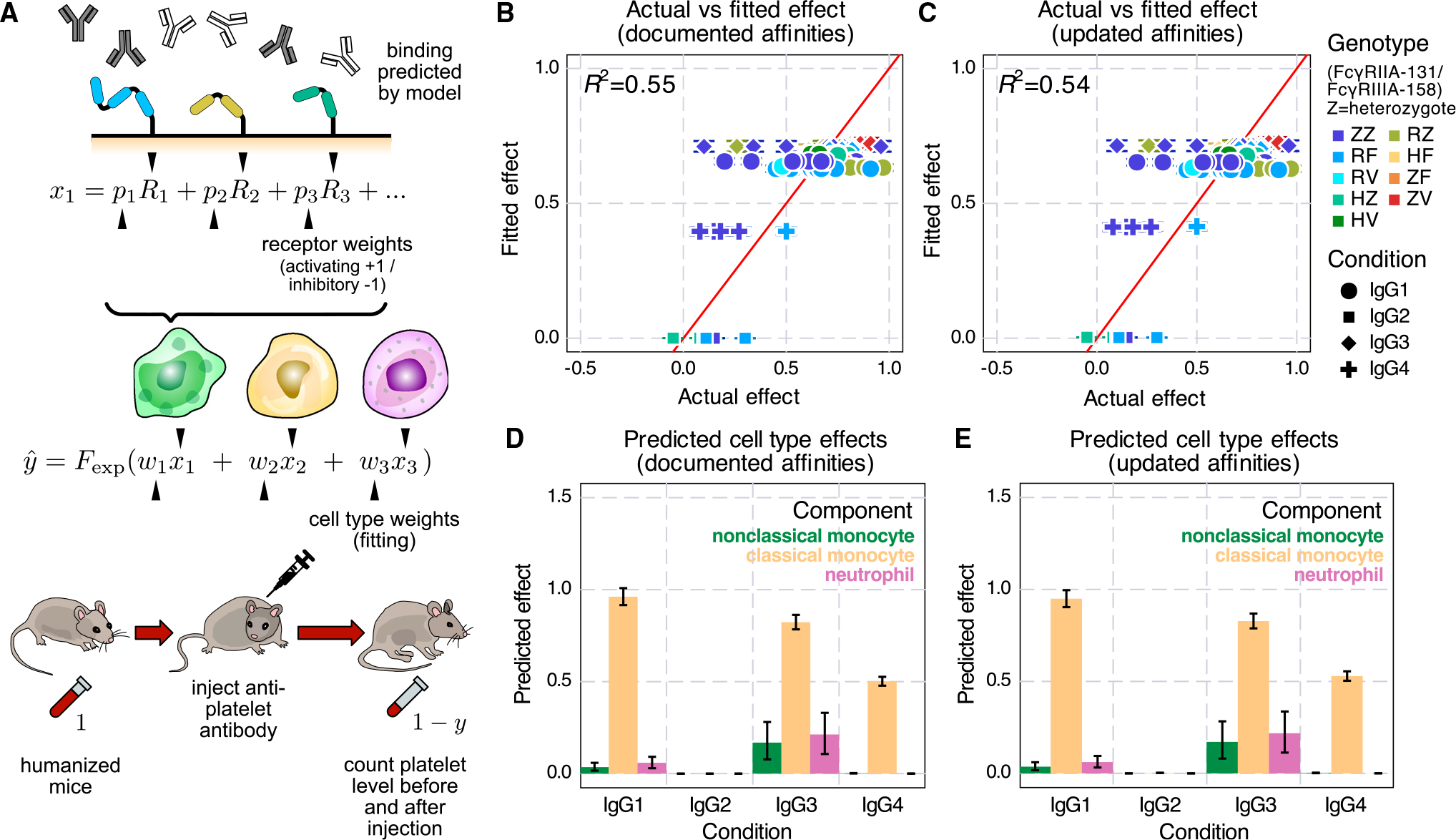
*In vivo* target cell depletion regression in humanized mice (A) Schematic of *in vivo* platelet depletion regression. To predict the percentage decrease of platelet abundance after antibody injection in mice, we combined the binding model predictions with the Fc receptor and effector cell type weights, then transformed the sum into depletion percentage with an exponential distribution cumulative density function. (B–E) Results of regression run using the documented (B and D) and updated (C and E) affinities. (B and C) Actual versus predicted depletion of platelets. (D and E) Predicted effector cell type effects. Error bars indicate the interquartile range from MCMC sampling.

Next, we used the multivalent binding model with regression to predict *in vivo* antibody effector cell-driven platelet depletion in humanized mice. In the process of extending our previous model, we elected to use the cumulative density function of the exponential distribution as the link function in our generalized linear regression model to link the overall cell activity to the amount of target (e.g., platelet) depletion (Figure 6A). Since the cell depletion effects have a limited range—one cannot deplete an antibody target of more than 100% or less than 0%—we must use a non-linear link function to transform the linear combination. While many functions provide this general relationship (such as the hyperbolic tangent function used before^16^), we realized that the extent of target cell depletion can be thought of as a form of survival analysis. In other words, given a certain antibody activity, a target cell has a certain probability of being cleared within the given timescale of the experiment. Assuming that all target cells have an equal propensity of being cleared dictates an exponential relationship for the link function.^21^

Having refined the cell clearance model, we applied it to a previously collected dataset examining *in vivo* platelet depletion in humanized mice.^22^ After fitting the cell type weighting, we found that the model fit the experiments well, especially considering the experiment-to-experiment variability due to donor graft variation and other sources of experimental uncertainty (Figures6B and 6C). The fitting was almost identical when using documented (Figure 6B) or updated (Figure 6C) FcγR affinities.

A benefit of the generalized linear regression model is that it provides an easy interpretation of each component. Inspecting the inferred cell type effects, we found that classical monocytes were inferred to be the predominant effector cell type (Figures 6D and 6E). IgG2 had some binding to each effector cell type, but no activity was inferred whatsoever (Figures 5F, 6D, and 6E). As the affinity updates are most relevant to IgG2, and this isotype had no *in vivo* effect, it is reasonable that these changes had little effect on agreement with the data (Figures 5F and 6E). While neutrophils, not classical monocytes, had the greatest binding, classical monocytes were inferred to exert the greatest impact on platelet depletion across isotypes (Figures 5A–5D). This demonstrates that the most bound cell type does not equate to the most potent effector. One explanation may be that, in these humanized models, there are relatively low numbers of human neutrophils upon reconstitution.^23^ The regression model can incorporate the molecular-level binding estimation and the depletion outcome to provide insights into the overall potency of each cell type. Overall, we found that the binding model could predict antibody-elicited effector responses *in vivo* in humanized mice.

## DISCUSSION

In this work, we explored the binding properties of ICs with mixed IgG Fc composition and linked their *in vitro* effects to *in vivo* effector cell-elicited platelet depletion. To quantify the binding of mixed IgG ICs *in vitro*, we measured every human IgG subclass pair across a range of compositions multimerized at two different valencies (Figure 1). Fitting these measurements to a model of multivalent interactions using documented affinities for each interaction, our model accurately captured the overall binding trends, with some outliers (Figure 3). We uncovered that the model discrepancies could be explained by inaccurate estimates of especially low-affinity Fc receptor interactions, most prominently involving IgG2. We validated revised affinities within an independent dataset and found that it greatly improved concordance with the data there as well. Finally, we used measurements of binding to effector cell populations to predict *in vivo* antibody-driven depletion of platelets in humanized mice (Figures 5 and 6). While the updated affinities did not change the agreement of the model with the observed depletion, it did change the interpretation of IgG2’s small effect on depletion—rather than not binding to classical monocytes, IgG2 binds strongly when in a larger IC, but platelets might provide insufficient avidity to observe sufficient engagement (Figure 6).

Considering that polysaccharide antigens present during bacterial infections or upon vaccination efficiently trigger IgG2 responses,^24^ our data would support the notion that FcγR-dependent effector functions such as phagocytosis of opsonized bacteria may contribute to protective IgG responses in humans more than expected. Conversely, autoreactive IgG2 responses observed during many autoimmune diseases may contribute to autoimmune pathology via FcγRs, which may warrant developing therapeutic interventions to block this pathway also in IgG2-dominated autoimmune diseases.^25^ Finally, with respect to the use of human IgG2 antibody formats as immunomodulatory antibodies for the therapy of cancer, our results would support strategies to engineer IgG2 variants with reduced binding to activating FcγRs and optimized binding for the inhibitory FcγRIIB, which has been shown to be critical for immunomodulatory IgG activity to further improve their therapeutic activity and reduce unwanted side effects.^26^

IgG subclasses and glycan variants are defined by their differing affinity toward each Fc receptor.^3,19,27^ Therefore, accurate measurements of each Fc receptor affinity are critical to understanding the differences in immune responses to each IgG. Using a mechanistic multivalent binding model alongside *in vitro* binding fluorescence measurements, we were able to derive a new set of Fc affinities refined from those measured by SPR. Due to the heightened avidity, multivalent ICs were better at detecting low-affinity IgG-Fc receptor interactions (Figure 4B). Examining binding through ICs also better simulates the relevant structure of Fc-FcR interactions *in vivo*. Harnessing avidity to overcome the low affinity of interactions is a common theme in immunology and its experimental characterization. For instance, tetramers are routinely used for isolating antigen-selective T cells.^28^ Here, we additionally show that these complexes can be used alongside quantitative models to infer properties of these systems.

This framework can be extended to study other aspects of IgG biology such as IgG allotypes. Due to a large number of variants and their implication in ADCC, IgG3 allotypes are of particular interest, with their immunogenicity directly related to their FcγR affinities.^29^ Compared with SPR, our multivalent strategy may allow us to better distinguish the subtle differences in allotype Fc affinities, while the binding model can predict their NK cell responses for different IC valencies. IgG polymorphism also presents in forms independent of Fc binding affinities, such as half-life and hinge length. Having a computational model that can accurately quantify the binding effect may help with separating the affinity-dependent and -independent factors, guiding optimal biologic designs.

Our results suggest that, within ICs comprised of several distinct subclasses or glycosylation variants, the Fc interaction effects are a blend of the constituent species’ properties. This means that ICs’ most extreme binding and effector responses should predominantly arise from whichever species is most potent in eliciting binding or a response. It also should provide some encouragement that the effector responses elicited from therapeutic antibodies should vary roughly in proportion to their relative composition; small contaminants of alternative Fc subclasses or glycosylation can only have a substantial effect if those species differ extremely in their responses alone. One caveat of this observation is that we only examined mixtures of antibodies with differing Fcs but identical antigen binding—polyclonal mixtures of antibodies will have still other interaction effects because antigens can form a higher-valency complex when they are present in combination.^30^ While in this work we only demonstrated Fc subclass mixtures, the same lessons likely apply to glycosylation mixtures, both *in vitro* and *in vivo*, since different subclasses and glycosylation variants exert their effect through divergent affinities toward Fc receptors.

Fc receptor-mediated effects are central to protection from both endogenously produced and therapeutic antibodies. Our work demonstrates that computational methods greatly facilitate reasoning about the complex signaling of the FcγR pathway quantitatively and at both cellular and organismal levels. This work extends our previous modeling to humanized mice and expands its application to the depletion of platelets.^16^ We anticipate that mechanistic models of antibody-mediated protection, such as the one here, will continue to grow in their utility for studying model systems such as humanized mice. In fact, as other features of antibodies are incorporated, such as variation in antigen specificity, it may become possible to connect behavior *in vitro* all the way to protection in human subjects.^31,32^

### Limitations of the study

Although the updated affinities performed better in predicting the binding to human lymphocytes, there were still discrepancies in the IgG4 predictions (Figures 5D, 5H, and 5J). The predicted binding to classical monocytes was higher than measured, especially with 4-valent complexes. This measurement might have been underestimated, as the measurement was close to 0, while the 33-valent binding was comparable to or higher than those of non-classical monocytes. IgG4-neutrophil binding was also underestimated. The most expressed FcγR on neutrophils is FcγRIIIB, but IgG4 was previously reported as non-binding to this receptor. Although they were not included in our subclass mixture study, from measurements of single IgG subclass complex binding to FcγRIIIB-expressing CHO cells, we inferred that the IgG2 and IgG4 affinities are both much lower than 10^5^ M^−1^, supporting the documented non-binding estimation (Figure S4; Tables S3 and S4). Alternatively, evidence exists that neutrophils also express a low level of FcγRIIIA, which has adequate binding to IgG4.^33^

To investigate the *in vivo* implication of our revised FcγR affinity updates, we elected to use humanized mice as a model system. This is both a strength and a limitation of this study. Humanized mice serve as an ideal surrogate for understanding human immunity.^34^ However, this model system is complicated by graft-to-graft differences, including the level of humanization and genetic heterogeneity of human stem cell donors.^34^ The depletion data reflected these complications, with high donor-to-donor and mouse-to-mouse variation, limiting our ability to observe subtle changes (Figures 6B and 6C).^22^

## STAR★METHODS

### RESOURCE AVAILABILITY

#### Lead contact

Further information and requests for resources and reagents should be directed to and will be fulfilled by the lead contact, Aaron S. Meyer (ameyer@asmlab.org).

#### Materials availability

All unique reagents generated in this study are available from the lead contact upon request.

#### Data and code availability

- The original data can be accessed in the “data” folder of the code repository.
- The original code is deposited on GitHub at https://github.com/meyer-lab/FcRegression.jl and on Zenodo at https://doi.org/10.5281/zenodo.7997263
- Any additional information request can be directed to the lead contact.

### EXPERIMENTAL MODEL AND STUDY PARTICIPANT DETAILS

#### Human participants

Aiming to investigate IC binding to primary human leukocytes, blood was drawn from seven healthy volunteers, six female and one male subject aged between 23 and 33 years, with the informed consent of the donors and the approval of the local ethical committee, Ethik-Kommission der Friedrich-Alexander-Universität Erlangen Nürnberg.

### METHOD DETAILS

#### Chinese hamster ovary (CHO) cell FcγR expression quantitation

Human FcγR expression on stably transfected CHO cells was quantified by determining the antibody binding capacity (ABC) for antibodies specific to the respective Fcγ receptor (Table S2).^13^ Quantum Simply Cellular (QSC) anti-mouse beads (Bangs Laboratories Ltd.) with known binding capacities for mouse IgG were used according to manufacturer’s instructions. Subsequently, a reference curve was generated by correlating the fluorescence intensity (caused by the respective anti-FcγR antibody) and the number of antibody binding sites of the different QSC beads. This reference curve was established in each experiment for all FcγR-specific antibodies of interest (PE-conjugated clone 10.1 to detect FcγRI, clone FUN-2 to detect FcγRIIA/B and clone 3G8 to detect FcγRIIIA, all from Biolegend) and used to calculate receptor numbers based on fluorescence intensity of FcγR staining on CHO cells. Samples were measured on a FACSCantoII flow cytometer and analyzed with FACSDiva software.

#### Immune complex binding measurement

CHO cells stably expressing human FcγRs were used to assess IgG-IC binding to hFcγRs as previously described.^6^ Briefly, ICs were generated by coincubation of 10 μg/mL anti-TNP human IgG subclasses (clone 7B4, produced in-house) and 5 μg/mL BSA coupled with either an average of 4 or 33 TNP molecules (Biosearch Technologies) to mimic low or high valency ICs, respectively, for 3 h with gentle shaking at room temperature. To address the impact of distinct subclass combinations on binding to hFcγRI, hFcγRIIA-131H/R, FcγRIIB and FcγRIIIA-158F/V, human IgG1 through IgG4 subclasses were mixed at specific conditions (100%, 90%, 66%, 33%, 10% of one subclass filled up to 10 μg/mL with the respective second subclass) before the addition of TNP-BSA. CHO cells stably expressing FcγRIIIB NA1 and NA2 variants were generated for this study and employed to determine IgG subclass binding for 100% IgG1–4 immune complexes of low and high valency. ICs were subsequently incubated with 100,000 FcγR expressing or untransfected control CHO cells for 1 h under gentle shaking at 4°C. Bound ICs were detected using a PE-conjugated goat anti-human IgG F(ab’)_2_ fragment at 0.5 μg/mL (Jackson ImmunoResearch Laboratories) on a BD FACSCanto II flow cytometer. To calculate the fluorescence signal intensity (median fluorescence intensity, MFI) of specific immune complex binding, the background fluorescence intensity of anti-human IgG F(ab’)_2_-stained control cells was subtracted (ΔMFI). The measured IC fluorescence intensities were between 1,000 and 15,000, far from the equipment saturation level which occurred at around 260,000. Each experimental condition had 3–5 technical replicates. The relative fluorescence unit of each IC binding was normalized so that measurements of each day had geometric means of 1.0.

Alternatively, binding to human primary peripheral blood leukocytes co-expressing specific FcγRs was studied. Blood was drawn from healthy volunteers and erythrocytes were lysed by the addition of ddH_2_O for 30 s at room temperature to obtain total leukocytes. Immune complexes were generated as described above and incubated with 200,000 leukocytes. Leukocyte subpopulations were identified by staining cell-type-specific surface markers. Fluorescently labeled antibodies PE/Cy7-conjugated anti-CD19, PerCP-conjugated anti-CD3, APC-conjugated anti-CD33, Brilliant Violet 510 conjugated anti-CD14, FITC-conjugated anti-CD56 and APC/Fire 750 conjugated anti-CD45 were obtained from Biolegend. Immune complex binding was quantified upon staining with PE-conjugated goat anti-human IgG F(ab’)_2_ fragment at 0.5 μg/mL (Jackson ImmunoResearch Laboratories) and data acquisition on a BD FACSCanto II flow cytometer.

The cell identification strategy was as follows: aggregates of cells were excluded by their forward light scatter (FSC) characteristics (area vs. height) and dead cells based on staining with DAPI. Leukocytes were identified by expression of common leukocyte marker CD45. Among those, neutrophils were gated based on high side light scatter (SSC) characteristics and lack of surface CD14, and classical monocytes were based on intermediate SSC and expression of CD14. Within the CD14^−^SSC^low^ cells, B and T cells were gated by expression of CD19 or CD3, respectively. Staining of CD56 was used to distinguish NK cells. The remaining CD33-expressing cells were gated as nonclassical monocytes. ΔMFI of bound immune complexes was calculated by subtracting the background fluorescence intensity of PBS-treated leukocytes.

Data were analyzed with FlowJo or FACSDiva Flow Cytometry Analysis Software. Six (6) biological replicates were measured for each IC valency, IgG subclass, and leukocyte cell type combination. All measurements were normalized so that the daily geometric means are 1.0.

### QUANTIFICATION AND STATISTICAL ANALYSIS

All statistical and computational analyses in this study were implemented by Julia v1.8.

#### Principal component analysis on mixture binding measurements

Principal component analysis on the IgG mixture binding measurement was performed with the package MultivariateStats.jl. The variance explained by principal component analysis was defined as $1-\frac{\|X-\hat{X}{\|}_{F}^{2}}{\|X{\|}_{F}^{2}}$ where $∥\cdot {∥}_{F}$ indicates the Frobenius norm.

#### Generalized multi-ligand, multi-receptor multivalent binding model

To model polyclonal antibody-antigen immune complexes (ICs), we employed a multivalent binding model to account for ICs of mixed IgG composition previously developed and detailed in Tan and Meyer.^20^

In this model, we define $N_{L}$ as the number of distinct monomer Fcs and $N_{R}$ as the number of FcRs, and the association constant of monovalent Fc-FcR binding between Fci and FcRj as $K_{a,ij.}$. Multivalent binding interactions after the initial interaction are assumed to have an association constant of $K_{x}^{*}K_{a,ij}$, proportional to their corresponding monovalent affinity. The concentration of complexes is $L_{0}$, and the complexes consist of random ligand monomer assortments according to their relative proportion. The proportion of ligand i among all monomers is $C_{i}$. By this setup, we know $\sum_{i=1}^{N_{L}}\;C_{i}=1.\;R_{\text{tot,}i}$ is the total number of receptors i expressed on the cell surface (where this term is used synonymously for the actual determined number of binding sites for the respective anti-FcR antibodies), and $R_{\text{eq,}i}$ the number of unbound receptors i on a cell at the equilibrium state during the ligand complex-receptor interaction.

The binding configuration at the equilibrium state between an individual complex and a cell expressing various receptors can be described as a vector $q=\left(q_{10},q_{11},\ldots ,q_{1N_{R}},\ldots ,q_{2N_{R}},\ldots ,\ldots ,q_{N_{L}N_{R}}\right)$ of length $N_{L}\left(N_{R}+1\right)$, where $q_{ij}$ is the number of ligand i bound to receptor j, and $q_{i0}$ is the number of unbound ligand i on that complex in this configuration. The sum of elements in q is equal to f, the effective avidity. For all i in $\left\{1,\;2,\ldots ,N_{L}\right\}$, let $\varphi_{ij}=R_{\text{eq,}j}K_{a,ij}K_{x}^{*}C_{i}$ when j is in $\left\{1,\;2,\ldots ,N_{R}\right\}$, and $\varphi_{i0}=C_{i}$. Then, the relative number of complexes in the configuration described by q at equilibrium is

$$v_{q,\text{eq}\;}=\left(\begin{array}{l} f\\ q \end{array}\right)\frac{L_{0}}{K_{x}^{*}}\prod_{i=1,j=0}^{i=N_{L,}j=N_{R}}\;\varphi_{ij}^{q_{ij}},$$

with $\left(\begin{array}{l} f\\ q \end{array}\right)$ being the multinomial coefficient. Then the total relative amount of bound receptor type n at equilibrium is

$$R_{\text{bound},n}=\frac{L_{0}f}{K_{x}^{\text{*}}}\sum_{m=0}^{N_{L}}\varphi_{mn}{\left(\sum_{i=1,\;j=0}^{i-N_{L},\;j=N_{R}}\varphi_{ij}\right)}^{f-1}.$$

By conservation of mass, we know that $R_{tot,n}=R_{eq,n}+R_{bound,n}$ for each receptor type n, while $R_{bound,n}$ is a function of $R_{eq,n}$. Therefore, each $R_{eq,n}$ can be solved numerically from its $R_{\text{tot},n}$ measured experimentally. Similarly, the total relative number of complexes bind to at least one receptor on the cell is

$$L_{\text{bound}}=\frac{L_{0}}{K_{x}^{*}}\left[{\left(\sum_{i=1,\;j=0}^{i=N_{L},\;j=N_{R}}\varphi_{ij}\right)}^{f}-1\right].$$

FcRs are activated through crosslinking. The amount of each kind of receptor in a multimerized complex can be calculated as

$$R_{\text{multi},n}=\frac{L_{0}f}{K_{x}^{*}}\sum_{m=1}^{N_{L}}\varphi_{mn}\left[{\left(\sum_{i=1,\;\;j=0}^{i=N_{L},\;j=N_{R}}\varphi_{ij}\right)}^{f-1}-1\right].$$

#### Immune complex binding analysis

Fitting the parameters in the binding quantification was performed by Markov chain Monte Carlo (MCMC) implemented by Turing.jl.^35^

At first, we plugged in the documented values into the binding model for all parameters without fitting, thus the geometric means of CHO cell receptor expression (Table S2), documented affinities,^3^ nominal valencies (4 and 33), and $K_{x}^{*}$ as 6:31 × 10^−13^cell·M^16^, as estimated in previous work (Figure 3C).^16^ To examine the role of affinity fitting, we used MCMC to fit all parameters except (Figure 3D) and including (Figure 3E) affinities. CHO receptor prior distributions were inferred from their measured values through maximal likelihood estimation (MLE) in Distributions.jl^36^ for both IgG mixture dataset (Table S2) and validation dataset^16^ (Table S5). The affinity priors were inferred from documented Fc affinities and standard errors following several assumptions: (1) each prior follows a log-normal distribution; (2) the mode of the distribution is the documented value, and the interquartile range of the distribution is the standard error; (3) if the values of mode or standard errors are too small, the mode was clipped to 1 × 10^4^ M^−1^, and the interquartile range was clipped to 1×10^5^ M^−1^ to deal with recorded nonbinding cases.^3,37^ The priors of the effective valency and crosslinking constant were:

$$f_{4}\sim \log\;N(\mu =\log(4),\;\sigma =0.2)$$

$$f_{33}\sim \log\;N(\mu =\log(33),\;\sigma =0.2)$$

$$K_{x}^{*}\sim \log\;N\left(\mu =\log\left(6.31\times 10^{-13}\right),\;\sigma =2.0\right)$$

MCMC was initialized with the maximum a posteriori estimation (MAP) optimized by a limited-memory BFGS algorithm implemented by Optim.jl,^38^ then sampled through a No U-Turn Sampler (NUTS) implemented by Turing.jl.^35^

#### *In vivo* regression model

We extended the *in vivo* antibody-elicited target cell depletion regression model with both cell type weights and FcγR weights (Figure 6A). Depletion, *y*, was represented as the percent reduction in the number of target cells.

To quantify the activity of each effector cell, we first used the multivalent binding model to predict the amount of multimerized FcγR of each kind, $R_{\text{multi},i}$, assuming each IC is 4-valent. Then the activity of this cell type is assumed to be a linear combination of these predictions and a set of receptor weights, $p_{i}$, that are set to either +1 or −1 for activating or inhibitory receptors, respectively, clipped to 0 if it is negative:

$$x_{n}=max\;\left(p_{1}R_{\text{multi,}1}+p_{2}R_{\text{multi,}2}+\ldots ,0\right)$$

To determine how these cell types bring the depletion effect at the organism level, we combine their estimated effects, $x_{n}$, with a weighted sum, where we introduce another set of weights, $w_{n}$, that are specific to each cell type. To convert the activities to a limited range of depletion (i.e., one cannot have a reduction over 100%), the regression was transformed by an exponential linker function (the cumulative density function of exponential distribution) such that the predicted effectiveness: $\overset{ˆ}{y}=F_{exp}(wx)=1-exp(-wx)$ so that ${lim}_{X\to \infty }\;F_{exp}(X)=1$. Together, we defined the estimated depletion as

$$\overset{ˆ}{y}=F_{exp}\left(w_{1}x_{1}+w_{2}x_{2}+\ldots \right)$$

We did not estimate the amount of each cell type in an individual, nor did we include them in the model, because the weights, $w_{n}$, are meant to absorb these quantities, while requiring effector cell abundance would limit the application of this model to organs where the tissue resident cell abundance has been accurately quantified.

The regression against *in vivo* effectiveness of IgG treatments was performed via MCMC implemented by Turing.jl.^35^ For the multivalent binding model, the ligand concentration was assumed to be 1 nM. The receptor expression level was set to the geometric means of the values measured in previous work (Table S6).^13^ For the receptor weights, $p_{i}$, we set the weight of the only inhibitory receptor, FcγRIIB, as −1:0 and every activating receptor to be + 1:0. The predicted cell type effects were estimated by multiplying the cell type weights by their predicted activities, $x_{n}$.

MCMC was initialized with MAP optimized by a limited-memory BFGS algorithm implemented by Optim.jl,^38^ then sampled through NUTS implemented by Turing.jl.^35^

## Supplementary Material

Supplementary information

## Figures and Tables

**KEY RESOURCES TABLE:** 

REAGENT or RESOURCE	SOURCE	IDENTIFIER

Antibodies		

Anti-human CD3 PerCP (mouse monoclonal)	Biolegend	Cat.#: 300428; RRID: AB_893298
Anti-human CD14 Brilliant Violet 510 (mouse monoclonal)	Biolegend	Cat.#: 301842; RRID: AB_2561946
Anti-human CD19 PE/Cyanine7 (mouse monoclonal)	Biolegend	Cat.#: 302216; RRID: AB_314246
Anti-human CD33 APC (mouse monoclonal)	Biolegend	Cat.#: 303408; RRID: AB_314352
Anti-human CD45 APC/Fire750 (mouse monoclonal)	Biolegend	Cat.#: 368518; RRID: AB_2616705
Anti-human CD56 FITC (mouse monoclonal)	Biolegend	Cat.#: 304604; RRID: AB_314446
Anti-human IgG Fc PE (goat Fab2 fragment)	Jackson Immunoresearch	Cat.#: 109–116-170; RRID: AB_2337681
Anti-human FcγRI PE (mouse monoclonal)	Biolegend	Cat.#: 305008; RRID: AB_314208
Anti-human FcγRIIA/B PE (mouse monoclonal)	Biolegend	Cat.#: 303206; RRID: AB_314338
Anti-human FcγRIIIA/B PE (mouse monoclonal)	Biolegend	Cat.#: 302008; RRID: AB_314208

Chemicals, peptides, and recombinant proteins		

TNP-4-BSA	BioSearch Technologies	Cat.#:T5050
TNP-33-BSA	BioSearch Technologies	Cat.#:T5050
Anti-TNP IgG1 (clone 7B4, human monoclonal)	Lux et al.^6^	In house
Anti-TNP IgG2 (clone 7B4, human monoclonal)	Lux et al.^6^	In house
Anti-TNP IgG3 (clone 7B4, human monoclonal)	Lux et al.^6^	In house
Anti-TNP IgG4 (clone 7B4, human monoclonal)	Lux et al.^6^	In house

Critical commercial assays		

Quantum Simply Cellular anti-mouse IgG	Bangs Laboratories	Cat.#: 815

Deposited data		

hIgG subclass mixture TNP-BSA IC binding to hFcγR-expressing CHO cells	This paper	https://github.com/meyer-lab/FcRegression.jl
hIgG pure subclass TNP-BSA IC binding to hFcγR-expressing CHO cells	Robinett et al.^16^	Figure 1
Residue platelet count in humanized mice 4 h after 6A6-hIgG injection	Schwab et al.^22^	Figure 1

Experimental models: Cell lines		

CHO	Lux et al.^6^	In house
CHO-hFcγRIA	Lux et al.^6^	In house
CHO-hFcγRIIA-131H	Lux et al.^6^	In house
CHO-hFcγRIIA-131R	Lux et al.^6^	In house
CHO-hFcγRIIB	Lux et al.^6^	In house
CHO-hFcγRIIIA-158F	Lux et al.^6^	M. Daeron, Institute Pasteur, Paris, France
CHO-hFcγRIIIA-158V	Lux et al.^6^	M. Daeron, Institute Pasteur, Paris, France
CHO-hFcgγRIIIB-NA1	This paper	In house
CHO-hFcγRIIIB-NA2	This paper	In house

**Software and algorithms**		

Julia	Julia Programming Language	https://julialang.org/
Multivalent binding model	Tan and Meyer^20^	https://github.com/meyer-lab/polyBindingModel.jl
Turing.jl	Ge et al.^35^	https://turing.ml/
Original source code	This paper	https://github.com/meyer-lab/FcRegression.jl
BDFACSDiva 6.1.3	Becton Dickinson, Franklin Lakes, NJ, USA	
FlowJo 10.8.1	FlowJo LLC, Ashland, OR, USA	
GraphPad Prism 9.5.1	GraphPad Software Inc, San Diego, CA, USA	

## Highlights

- Mixed Fc immune complexes bind as a blend of each constituent IgG species
- A multivalent binding model can be generalized to mixed Fc immune complexes
- Immune complexes provide better estimates for low IgG-Fcγ receptor affinities
- The FcγR binding model predicts effector-elicited cell clearance in humanized mice
